# Fibrosis: a distinguishing feature in the pathology of neural leprosy

**DOI:** 10.1590/0074-02760190056

**Published:** 2019-08-05

**Authors:** Sérgio Luiz Gomes Antunes, Márcia Rodrigues Jardim, Robson Teixeira Vital, Bernardo Miguel de Oliveira Pascarelli, José Augusto da Costa Nery, Thaís Porto Amadeu, Anna Maria Sales, Eduardo Alves Freire da Costa, Euzenir Nunes Sarno

**Affiliations:** 1 Fundação Oswaldo Cruz, Instituto Oswaldo Cruz, Laboratório de Hanseníase, Rio de Janeiro, RJ, Brasil; 2 Universidade do Estado do Rio de Janeiro, Departamento de Patologia e Laboratórios, Rio de Janeiro, RJ, Brasil

**Keywords:** leprosy, neuropathy, fibrosis

## Abstract

**BACKGROUND:**

Fibrosis in the peripheral nerve is the end stage of leprous neuropathy and the cause of the resulting permanent neural function impairments. Preventive measures to avoid this irreversible pathological state are a relief strategy for leprosy sufferers.

**OBJECTIVES:**

The present study describes the frequency of fibrosis along with its characterisation and pathogenic development.

**METHODS:**

Six-hundred-and-thirteen nerve samples were sorted from 278 neural leprosy (NL) and 335 non-leprosy neuropathy patients (ON). The total number of samples was histologically examined by routine staining methods (haematoxylin-eosin, Wade staining and Gomori’s trichrome) and fibrosis was evaluated via semi-quantitative estimation.

**FINDINGS:**

Fibrosis was most frequent in the NL group (33% against 0.4% in ON) while fibrosis in association with endoneurial microfasciculation was found in 38 (41.3%) of the NL samples in the examination of semithin sections. Pericytic activation in the perivascular environment was confirmed to be the source of the fibroblasts and perineurial cells delimiting microfascicles. End-stage fibrosis in leprosy displays an arrangement of microfascicles devoid of neural components (i.e., Schwann cells and axons) lined by an intermediate phenotype of fibroblastic-perineurial cells filled with bundles of collagen fibres.

**MAIN CONCLUSIONS:**

The present study underscores that fibrosis is frequently the severe end stage of neural leprosy NL pathogeny after analysing the notably distinct development of fibrosis within the neural environment.

Leprosy predominantly affects the skin and peripheral nervous system and, less frequently, other organs such as the upper respiratory tract, bones and eyes.^1^ Although leprosy has a low mortality rate, peripheral neuropathy is the main cause of patient morbidity, often leading to the disabilities and deformities commonly associated with the disease^2^.

In leprosy, injury in the peripheral nervous system is characterised by either an inflammatory infiltrate that may be highly activated and exhibit epithelioid granulomas or a hyporesponsive infiltrate to *Mycobacterium leprae* that displays an accumulation of foamy macrophages loaded with acid-fast bacilli (AFB) interspersed by lymphocytes and plasma cells.^3^ The most commonly-affected segments of the peripheral nervous system are the nerve trunks and branches, as well as their endings in the skin. The inflammatory process extends across the nerve compartments, causing a severe loss of large and small myelinated fibres along with compromised non-myelinated fibres, resulting in denervated Schwann cells.^3^ ^,^ ^4^ All these structural alterations lead to impairments in the sensory and motor functions. This pathogenic process evolves until achieving nerve destruction and fibrosis. In turn, fibrosis replaces the affected nerve fibres by transforming them into a fibrous string, resulting in blurring the histological boundaries between the perineurial and endoneurial compartments.^4^ Nerve fibrosis is an irreversible condition that should be diligently avoided due to the ensuing permanent disabilities and physical deformities suffered by these patients. 

According to Antunes et al.,^4^ fibrosis in NL is unique in that, in the samples examined, no other neuropathy exhibited such a high degree of collagen deposit density. The finding of fibrosis in the histological examination of biopsy nerve specimens, together with the clinical, electroneuromyographic and laboratory alterations (polymerase chain reaction - PCR) gleaned from suspected leprosy patients, is plausible indications of a probable leprosy diagnosis in AFB nerve samples. These criteria are particularly strengthened in highly prevalent countries for leprosy. The presence of fibrotic dermal nerve branches is also regularly detected in the cutaneous biopsy specimens collected from the often apparently normal skin of PNL patients.^5^

Vallat et al.^6^, followed by Antunes et al.,^4^ described a peculiar histopathological aspect of NL characterised by the presence of microfascicles having an increased collagen fibre content having been formed by an abnormal distribution of fibroblastic cells surrounding groups of small myelinated and non-myelinated fibres together with denervated Schwann cells. Vallat et al.^6^ considered this event as a proliferation of the perineurial cells simultaneously engaged in invading the endoneurium and intervening in the remaining nerve fibres surrounding them to form the aforementioned microfascicular nest-like structures. According to Antunes et al.^7^ first report, in the histopathological examination of NL, the frequency of miscrofasciculation was low (10.9%). Besides, it was only found in the leprosy nerve samples and not in the ones of non-leprosy neuropathies.

In summary, the present descriptive study reveals the real dimensions of fibrosis in NL along with a detailed overview of its morphological characteristics and frequency rate. The peculiar fibrogenic process underway in NL has been closely analysed to demonstrate the interactions among the endoneurial fibroblasts, perineurial cells, pericytes, Schwann cells and microfascicles that contribute to the final, irreversible fibrotic configuration of the nerves in leprosy disease.

## MATERIALS AND METHODS

Histological sections of nerve samples collected from 278 NL patients as well as 335 with non-leprosy neuropathies (ON) were selected for the present study (Table). All patients were then in treatment at the Ambulatório Souza Araújo of the Instituto Oswaldo Cruz (IOC) (Souza Araújo Outpatient Service of the Oswaldo Cruz Institute), Rio de Janeiro, state of Rio de Janeiro, Brazil. Their clinical, electroneurophysiological and laboratory data (bacilloscopy, histology of nerve biopsies and nerve-sample detection of *M. leprae* DNA) were routinely collected for diagnosis and follow up. Investigative laboratory tests were conducted to confirm a leprosy diagnosis in the context of highly suggestive clinical and laboratory patterns of peripheral neuropathy in a prevalent region of Brazil.

**TABLE:** Frequency of fibrosis and microfasciculation in leprous nerve samples (NL) and other neuropathies (ON)

Total of 613 nerve samples	
NL	278 (45.3)
ON	335 (54.6)
NL (278 samples)	
Pure NL (PNL)	190 (68.3)
Post-treatment leprosy neuropathy (PTLN)	88 (31.6)
Fibrosis in 278 NL samples	
Increased extracellular matrix	123 (44.2)
Endoneurial fibrosis	92 (33)
Normal extracellular matrix	63 (22.6)
Microfasciculation	
In 278 NL samples	36 (12.9)
In 92 NL samples showing fibrosis	38 (41.3)
Fibrosis in 335 ON samples	
Increased extracellular matrix	52 (15.5)
Fibrosis	3 (0.4)

Data presented as n (%).

The NL group of samples (278) consisted of 190 from patients with pure NL (PNL) and 88 from those with post-treatment leprous neuropathy (PTLN). Diagnoses of the PNL patients were confirmed or not according to Jardim et al.^8^ and Antunes et al.^4^ guidelines.

The PTLN group of samples was composed of patients who had symptoms of post-treatment leprosy neuropathy, were refractory to anti-reactional treatment and presented worsening impairment of their neural functions. This clinical picture tends to imply that the PTLN group is made up of refractory reactional patients that could be undergoing a relapse of infection, reinfection or an intervening non-leprosy neuropathy. To carry out reliable differential diagnoses, nerve biopsies are necessary. All these patients completed their multidrug treatment (MDT) more than five years prior to the diagnostic nerve biopsy taken at the time of the present study.

The ON tag was assigned to those whose test results proved to be inconsistent with leprosy neuropathy. Once determined that a PNL diagnosis could not be confirmed, the ON patients were forwarded to specialised clinical services for diagnosis and follow-up. It was diagnostically confirmed that many among the 335 ON patients had diabetic neuropathy, amyloidosis, alcoholic neuropathy, chronic inflammatory demyelinating polyneuropathy, human immunodeficiency virus (HIV) neuropathy or compression neuropathy.

The nerves selected for biopsy procedures were the dorsal cutaneous branch of the ulnar, sural, superficial fibular, radial and median nerves.

All of the nerve samples were divided into two parts. Part 1 was processed by routine histopathological examination, fixed in 4% paraformaldehyde, dehydrated, clarified with xylene and embedded in paraffin. Paraffin blocks were sectioned at a microtome (Thermo Fisher Scientific - Thermo Shandon, Massachusetts, USA) into 5 μm-thick sections. The sections were laid on glass slides, had their paraffin removed via serial xylene and alcohol immersion, were rehydrated and then stained with haematoxylin-eosin to evaluate the inflammatory infiltrate and cell populations by using Gomori’s trichrome to assess fibrosis and nerve structure and Wade staining to detect AFB. Part 2 of the nerve samples was fixed in 2.5% glutaraldehyde, washed in sodium cacodylate buffer, post-fixed in 2% osmium tetroxide, washed again in the same buffer, dehydrated in serial-graded acetone batches, impregnated and included in epon. The blocks were sectioned into 0.5-μm-thick sections (ultramicrotome from Reichert, New York, USA) and stained in toluidine blue

**Fig. 1:**
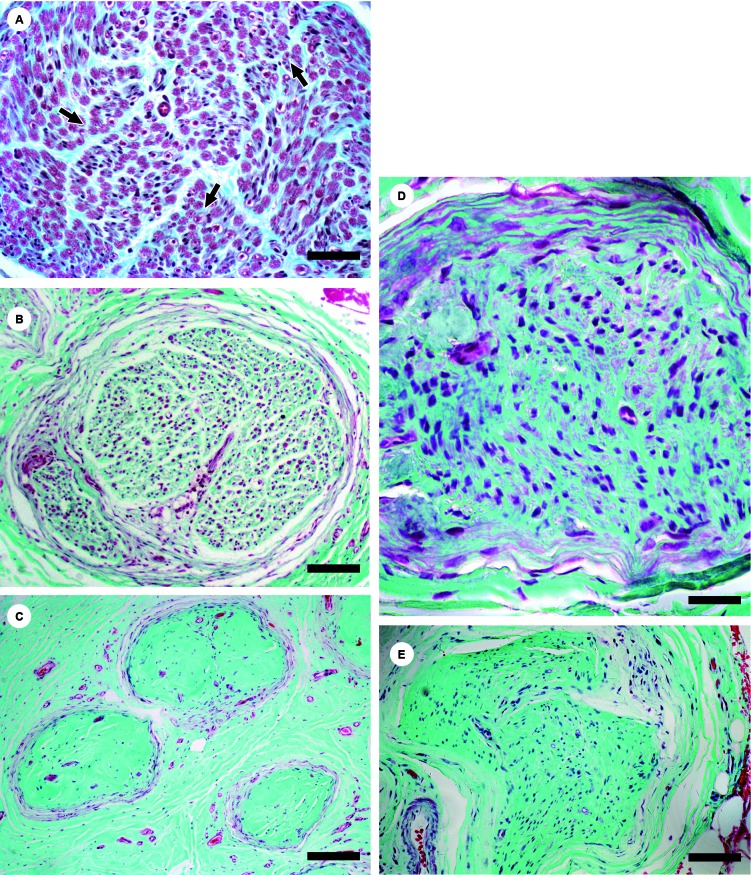
A: green-stained percentage rate lower than 40% (normal nerve); myelin sheaths of nerve fibres are stained in red (arrows). Bars: 70 µm; B: extracellular matrix percentage rate between 40 and 60% increased extracellular matrix in a PNL nerve - note the decreased number of fibres. Bars: 140 µm; C: extracellular matrix percentage rate above 60% (fibrosis in a PNL nerve). Bars: 250 µm; D: extracellular matrix percentage rate between 40 and 60% increased extracellular matrix in other neuropathies (ON) nerve. Bars: 70 µm; E: extracellular matrix occupation rate above 60% (fibrosis in ON nerve). Bars: 140 µm.

Fibrosis was measured via Gomori’s trichrome staining by employing a semi-quantitative estimation of the percentage of light-green-stained endoneurium in the fascicular cross section area. The degree of fibrosis was assigned according to the following criteria: 0 = absence of fibrosis; grade 1: increased extracellular matrix; grade 2: presence of fibrosis (Fig. 1C). Absence of fibrosis (grade 0) corresponds to a light-green stained endoneurium of up to 40% of the endoneurial fascicular cross section area (Fig. 1A); increased extracellular matrix (grade 1) represents the light-green-stained endoneurium covering between 40 and 60% of the fascicular cross section area (Fig. 1B, D). This percentage did not reach the level of fibrosis as reduction in the quantity of nerve fibres caused increased stromal occupation of the endoneurial compartment. In these cases, the presence of cells amid the increased extracellular matrix indicated that fibrosis had not yet been fully established. Finally, the presence of fibrosis was determined if the percentage of light-green-stained endoneurium covered more than 60% of the endoneurial cross section area (Fig. 1C, E). Light green is the colour of the dye used in Gomori’s trichrome staining procedure. In addition, density and homogeneity of the extracellular matrix were factored in when evaluating fibrosis.

*Immunohistochemistry -* Sections from five nerve samples presenting endoneurial microvessels accompanied by activated pericytes were deparaffinised with xylene and rehydrated in a series of graded ethanol, ranging from 100 to 70%, and a phosphate-buffered saline (PBS) (0.01, pH 7.4). Inhibition of endogenous peroxidase was performed with 3% H202 followed by a Dako antigen retrieval (Dako Corp., Carpinteria, CA, USA) application for 15 min at 90°C. Non-specific antibody binding was blocked by 10% nonimmune serum [from goat serum for anti-nerve growth factor receptor (NGFr), anti-α-smooth muscle actin (α-sma) and anti-CD34] for 1 h at room temperature (RT) and then removed by tipping the drop off the slides.

The primary antibodies utilised were mouse anti-NGFr (1/50) (Agilent Dako, Santa Clara, USA) mouse anti-CD34 (1/50) (Dako, USA) and mouse anti-α-sma (1/200) (Dako) diluted in Tris-buffered saline, pH 7.4 (Sigma-Aldrich, Missouri, USA). The primary antibodies were rinsed with PBS after which goat anti-mouse second biotinylated antibodies (1:100) (Dako) were applied. Both secondary antibodies were diluted in 0.01M PBS added to 2% nonimmune goat serum (Dako). The sections were incubated with the avidin-biotin complex (VectaStain ABC kits; Vector Laboratories, Burlingame, CA, USA) for 1 h at RT. Immunostaining was developed in a solution containing diaminobenzidine (Sigma) and hydrogen peroxide, counterstained with Meyer’s haematoxylin, dehydrated in graded alcohol, clarified in xylene and mounted with Entellan medium (Merck, Darmstadt, Germany). Control sections incubated with non-immune goat serum and another slide incubated with only diaminobenzidine (Sigma) were utilised as specificity and endogenous peroxidase controls, respectively.

All the sections were examined under a Nikon Eclipse E400 optical microscope (Minato, Japan) . Semithin sections in this study were employed to be able to visualize the very delicate cytoplasmic processes within fibroblastic cells amid the dense extracellular matrix deposit involved in the neural fibrotic process, rarely visible in paraffin-embedded specimens.

The frequency of fibrosis in the total number of nerves examined was determined as well as the presence of microfascicles and their association with nerve fibrosis in leprosy.

This work was approved by the Internal Board Review of the IOC according to statement 2.022.517

## RESULTS

In the NL group (278 samples), 123 (44.2%) samples exhibited increased extracellular matrix and 92 (33%) presented fibrosis in accordance with the criteria cited in Materials and Methods. Sixty-three (22.6%) NL samples had no signs of fibrosis in the histological examination whereas histopathological images of semithin sections from 38 leprosy samples exhibiting fibrosis revealed the presence of microfascicles filled with bundles of collagen instead of nerve fibres (Table).

Just three samples in the ON group (0.4%) exhibited fibrosis (Fig. 1E) and 52 (15.5%) showed a relatively increased light-green-stained area due to loss of myelinated fibres and consequent occupation of this area by connective tissue (Fig. 1D). Absent in the ON group were the other characteristics that often accompany fibrosis in leprosy such as an hyalinisation of the endoneurium, a dramatic decrease in cell population, and the blurring of nerve compartment boundaries (Table).

**Fig. 2:**
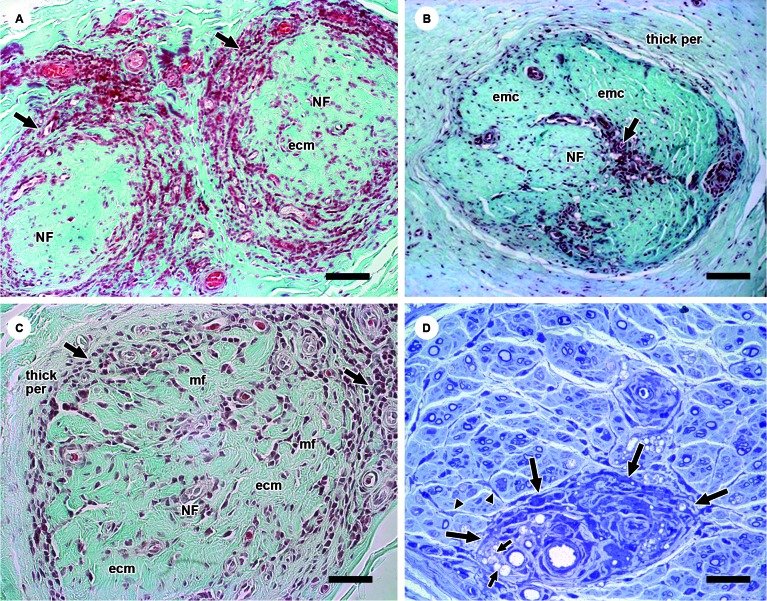
A: two leprosy-affected nerve fascicles (NF) display a dense perineurial collar of mononuclear inflammatory cells (arrows). Perineurial cells are absent after being destroyed by the infiltrate. The fascicles are filled with dense, green-stained extracellular matrix (ecm) and residual inflammatory cells. Gomori’s trichrome. Bars: 160 µm; B: a NF exhibiting increased green-stained, homogeneous ecm permeated by mononuclear cell infiltrate composed of lymphocytes and foamy macrophages (arrow). The perineurium is thickened (thick per) due to excessive extracellular matrix and an increased number of perineurial layers. Gomori’s trichrome. Bars: 160 µm; C: a NF exhibiting a perineurial collar of mononuclear inflammatory cells (arrow) delimiting an endoneurial compartment with increased ecm) An incipient microfascicle formation (mf) and a thickened perineurium (thick per) are shown. Gomori’s trichrome. Bars: 80 µm; D: a NF showing a perivascular inflammatory focus (delimited by long arrows) consisting of foamy macrophages (short arrows), lymphocytes, activated pericytes and fibroblasts. This microfocus gives origin to fibroblasts that irradiate to the circumjacent endoneurium (arrowheads). Semithin section, toluidine blue staining. Bars: 80 µm.

**Fig. 3:**
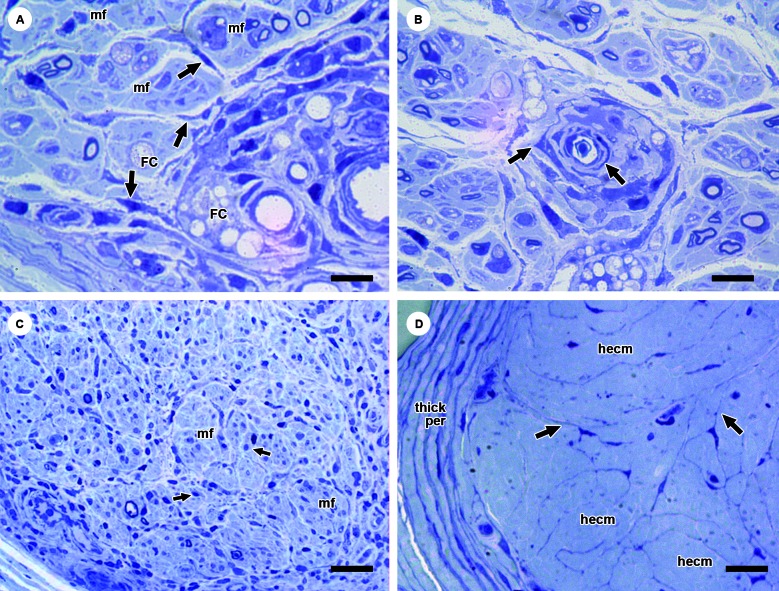
A:close view of the perivascular region of Fig. 2D showing the inflammatory infiltrate surrounding the endoneurial microvessel. Elongated fibroblasts (arrows) can be seen spread throughout the stromal interstitium to the circumjacent endoneurial environment, initiating the formation of microfascicle (mf)-containing cells. Foamy cells (FC) are also clearly visible. Semithin section, toluidine blue staining. Bars: 18 µm; B: prominent endoneurial capillary vessel of a leprosy-affected nerve exhibiting a higher number of concentric layers of pericytes surrounding the vascular structure (arrows). Semithin section, toluidine blue staining. Bars: 18 µm; C: leprosy-affected nerve fascicle showing an endoneurial compartment occupied by microfascicles (mf) whose contents display small, residual myelinated fibres and sparse mononuclear cells (short arrows). Fibrosis is not yet present. Semithin section. Toluidine blue staining. Bars: 40 µm; D: end stage of a leprosy-affected nerve fascicle exhibiting slight perineurial thickening (thick per) and a mf-filled endoneurial compartment. The contents of the mf are devoid of cells, which were replaced by an exclusive hyaline extracellular matrix (hecm). Note the slender cytoplasmic processes of the fibroblastic-perineurial cells (arrows). Semithin section. Toluidine blue staining. Bars: 40 µm.

*Description of the histological characteristics of fibrosis in leprosy nerves -* Most of the samples showed nerve fibre loss due to the pathogenic mechanism of leprosy (*M. leprae*-induced axonal demyelination and degeneration as a result of the endoneurial inflammatory process)^4^ (Figs. 2A-D, 3C, D). As the quantity of nerve fibres decreased, the endoneurial extracellular matrix in the histological sections replaced the existing nerve fibres (Figs. 2A-C). Fibrosis was also detected in the perineurial compartment under the guise of a relatively increased extracellular matrix (green-stained area) and a greater number of perineurial cell layers (Figs. 2B, C, 3D).

The boundaries between the perineurium and epineurium were blurred due to the excessive extracellular matrix deposit, which, over time, became relatively homogeneous and hyalinised (Fig. 2C). In the more severely fibrotic samples, the recognition of nerve fascicles was challenging. The cell population dramatically decreased in the endoneurium and those that remained were either fibroblasts, scarce residual mononuclear inflammatory cells or newly-formed capillary vessels. The endoneurium became devoid of neural structures but full of collagen fibres contained in the microfascicles delimiting the fibroblastic perineurial cells (Fig. 3D).

A pattern of microfasciculation was noticed in 12.9% of the NL biopsy samples (Figs. 2D, 3A, C, D). At the same time, the association with fibrosis was high in the nerve specimens exhibiting fibrosis (41.3%) (Fig. 3D). This pattern was more easily recognisable in the semithin sections (0,5-µm-thick), in which the thin cytoplasmic processes of endoneurial fibroblasts were more sharply visualised than in the 5-µm-thick sections obtained from the paraffin-embedded blocks (Fig. 3D).

In the initial stages, microfascicles contain small myelinated and non-myelinated fibres, denervated Schwann cells, inflammatory cells and collagen fibres. Content identification would only have been possible by way of transmission electronmicroscopy, unavailable in the present study. As the degree of fibrosis advances, microfascicles become progressively collagenised and devoid of axons and Schwann cells. 

To reiterate, the fibrotic nerve is assembled as a group of microfascicles devoid of nerve components and delimited by the fibroblastic-perineurial cells (Fig. 3D). Morphological evidence shows that the fibroblastic-perineurial cells outlining microfascicles are derived from the activated pericytes surrounding the endoneurial microvessel lumen shown in Fig. 3B. Pericytes differentiate into fibroblasts, which then give origin to perineurial-fibroblastic cells that end up encircling the microfascicles (Fig. 3A, B)

**Fig. 4:**
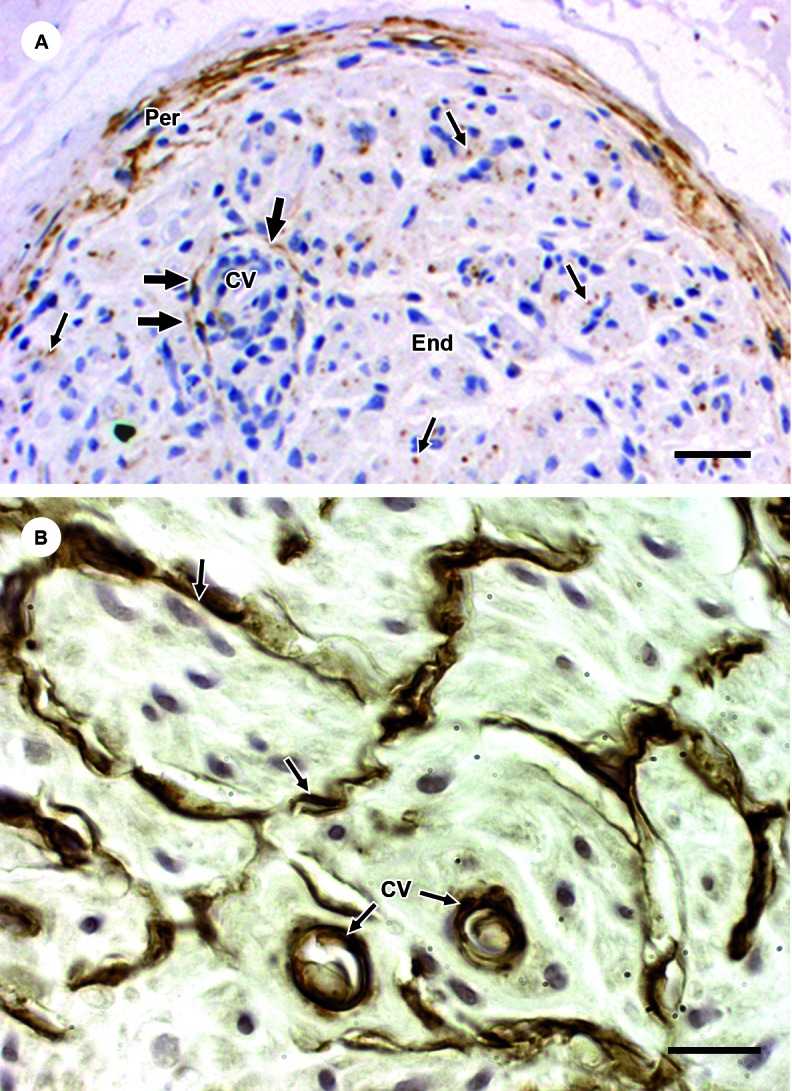
A: a leprosy nerve fascicle evidencing the endoneurium (End) and perineurium (Per). The image showing NGFr-immunoreactive fibroblastic cells (thick arrows) surrounding an endoneurial capillary vessel (cv) of a leprosy nerve sample strongly suggests that pericytes are the source of the fibroblastic-perineurial cells that form microfascicles. Perineurial cells (Per) and small axons (thin arrows) express nerve growth factor receptor (NGFr). Immunoperoxidase staining. Bars: 60 µm; B: CD34-immunoreactivity in the perineurial cells (arrows) lining the microfascicles demonstrates that the perineurial cells share a fibroblastic-phenotypic feature. Capillary endothelial cells (cv with thin arrows) are CD34-immunoreactive. Immunoperoxidase avidin-biotin system. Bars: 50 µm.

NGFr-immunoreactive fibroblastic cells were seen in the perivascular inflammatory environment of NL samples (Fig. 4A). No α-sma-immunolabelled fibroblast-like cell was identified in the endoneurial compartment. Perineurial-fibroblastic cells outlining the microfascicles share both the CD34-fibroblastic phenotype (Fig. 4B) and NGF-perineurial cell expression (Fig. 4A).

## DISCUSSION

The present study confirmed the evidence that nerve fibrosis is a relevant feature in leprosy neuropathy. The ON group, used as a comparative control, presented a very low frequency of fibrosis (0.4%) while the increased extracellular matrix was due to loss of myelinated fibres. No hyalinisation of the endoneurium nor any blurring of the boundaries among the endoneurium, perineurium and epineurium were seen in the ON group. It is known that nerve fibrosis is found in traumatic and post-radiation neuropathy.^9^ ^,^ ^10^ These aetiologies were not confirmed in the ON group.

In the present work, however, it was not possible to ascertain the causes for this frequent and peculiar fibrotic presentation in NL. Nonetheless, it can be speculated that the imbalance between the fibrogenic and anti-fibrogenic factors specifically induced during the leprosy pathogenic process is a probable cause. As expected, CCL2, a chemokyne involved in such fibrogenic diseases as systemic sclerosis,^11^ increased its expression in Schwann cells and in the infiltrating macrophages of leprosy-affected peripheral nerves.^12^ In this connection, Athaide et al.^13^ reported an increased *in vitro M. leprae*- and transforming growth factor (TGF)β-induced expression of platelet-derived growth factor (PDGF)-BB, in addition to their receptors PDGFR α and β, on the ST88-14 Schwann cell lineage. It is known that PDGF and its isoforms are involved in scar formation and the pathogenesis of fibrosis in diverse organs.^14^ ^,^ ^15^ These findings could be a clue to explaining the peculiar fibrotic pattern of NL at its end stage.

The repair process in endoneurial stroma is distinct from that observed in non-neural connective tissue, as endoneurial fibroblasts show phenotypic, morphological and behavioural features different from their corresponding cells in non-neural stroma. According to Richard et al.,^16^ the endoneurial fibroblast family is composed of distinct interrelated subsets (fibroblasts, pericytes, and interstitial Cajal cells) that could be the source of fibroblastic-perineurial cells.^17^ In addition to this complexity, Schwann cells are capable of transdifferentiating into myofibroblasts^18^ ^,^ ^19^ while fibroblasts give origin to perineurial cells.^20^

The morphological examination of the leprous nerve biopsy specimens in the present study frequently disclosed activated pericytes surrounding endoneurial microvessels involved in inflammatory foci. Morphological evidence of activation is represented by the increased size of pericytes, formation of concentric hyperplastic pericytic layers surrounding microvessels and the morphological impression of spreading given by a noticeably decreasing concentration gradient of fibroblastic cells from the perivascular region to the circumjacent endoneurium through the endoneurial stromal septa (Fig. 3A, B). The evidence referred to above was detected in the specimens in which fibrosis was still absent or perhaps incipient, clearly indicating that pericytes could be the source of fibroblasts. The presence of NGFr-immunoreactive fibroblastic cells in the perivascular endoneurial environment involved in the leprous inflammatory process and the pluripotent activity of pericytes^21^ are sure signs that pericytes are a source of the fibroblasts that gradually differentiate into perineurial cells (Fig. 3D). 

The frequent observation of fibrosis and microfasciculation in NL^4^ ^,^ ^7^ ^,^ ^9^ led us to determine a relationship between these two findings in NL samples with fibrosis. An association between fibrosis and the presence of collagen-filled microfascicles was clearly observed in the semithin sections.

It is worth commenting that, in the present study, microfasciculation was more frequent than in the previous Antunes et al.^7^ report, perhaps due to the visualisation of the fibroblastic-perineurial cells lining the microfascicles, enabled by the histopathology of semithin sections. The above-cited method made it possible to demonstrate an association between fibrosis and microfasciculation and determine that the latter preceded massive fibrosis of the nerves.

Microfasciculation was more frequent in multibacillary nerves in which the pace of destruction is slower and the nerve stromal structure remains relatively preserved compared to what occurs in rapidly-damaging paucibacillary tuberculoid granulomas.

Antunes et al.^7^ had also depicted the existence of an intermediate phenotype between perineurial (NGFr-immunoreactivity) and endoneurial fibroblastic cells (CD34-immunolabeling together with lack of a basement membrane) (Fig. 4A) of the perineurial cells lining microfascicles. Therefore, in face of this morphological observation, pericytes, fibroblasts and perineurial cells came to be considered parts of the sequential cellular stages in the leprous fibrotic process.

It is worth pointing out here that, during development and in the adult response to injury, Schwann cells orchestrate nerve restructuring by modulating perineurial thickness, forming nerve fascicles, organising the nerve stroma, establishing a blood-nerve barrier; and regulating Schwann cell-axon interaction.^22^ ^,^ ^23^ The role of Schwann cells is mediated by the desert hedgehog factor (Dhh), a cytokine involved in peripheral nerve ontogeny.^24^ Consequently, the progressive disappearance of Schwann cells in leprosy-affected nerves creates a permissive environment for the massive collagenisation of endoneurial microfascicles in NL effected by the lining of microfascicles by fibroblastic-perineurial cells.

Again, in the present study, genetic reprogramming of Schwann cells generating myofibroblasts or other multiple matrix-producing mesenchyme-derived cells^19^ ^,^ ^25^ was not found to be relevant to the mechanisms related in fibrosis in NL. Moreover, no α-sma was detected via the immunohistochemical method employed.

In conclusion, severe fibrosis is a frequent event in the final stage of leprosy neuropathy, causing irreversible structural and functional damage to the nerves. It is significantly more frequent in NL than in non-leprosy peripheral neuropathies.

Severe fibrosis was associated with the pericytic differentiation into fibroblasts and, later, into perineurial cells that eventually surround and delimit microfascicles. The fibroblastic-perineurial cells lining the microfascicles are the collagen-producing agents in NL fibrosis. The primary or effective cause of fibrosis has yet to be determined. 

It is hoped that the present study contributes to promoting the development of a pathway for future interventions to effectively prevent the development of permanent disabilities in leprosy neuropathy.
